# The salivary proteome in relation to oral mucositis in autologous hematopoietic stem cell transplantation recipients: a labelled and label-free proteomics approach

**DOI:** 10.1186/s12903-023-03190-w

**Published:** 2023-07-07

**Authors:** S. J. M. van Leeuwen, G. B. Proctor, A. Staes, A. M. G. A. Laheij, C. M. J. Potting, M. T. Brennan, I. von Bültzingslöwen, F. R. Rozema, M. D. Hazenberg, N. M. A. Blijlevens, J. E. Raber-Durlacher, M. C. D. N. J. M. Huysmans

**Affiliations:** 1 Department of Dentistry, Radboud University Medical Center, Nijmegen, The Netherlands; 2 Centre for Host Microbiome Interactions, King’s College London Dental Institute, London, UK; 3 VIB Proteomics Core, VIB Center for Medical Biotechnology, Ghent, Belgium; 4 Department of Biomolecular Medicine, Ghent University, Ghent, Belgium; 5 Department of Oral Medicine, Academic Centre for Dentistry Amsterdam, University of Amsterdam and VU University, Amsterdam, The Netherlands; 6 Department of Preventive Dentistry, Academic Centre for Dentistry Amsterdam, University of Amsterdam and VU University, Amsterdam, The Netherlands; 7 Department of Oral and Maxillofacial Surgery, Amsterdam UMC, University of Amsterdam, Amsterdam, The Netherlands; 8 Department of Hematology, Radboud University Medical Center, Nijmegen, The Netherlands; 9 Department of Oral Medicine/Oral and Maxillofacial Surgery, Atrium Health Carolinas Medical Centre, NC Charlotte, USA; 10 Department of Otolaryngology/Head and Neck Surgery, Wake Forest University School of Medicine, NC Winston-Salem, USA; 11 Department of Oral Microbiology and Immunology, Institute of Odontology, The Sahlgrenska Academy, University of Gothenburg, Gothenburg, Sweden; 12 Department of Hematology, Amsterdam UMC, University of Amsterdam, Amsterdam, The Netherlands; 13 Department of Hematopoiesis, Sanquin Research, Amsterdam, The Netherlands

**Keywords:** Oral mucositis, Saliva, TMT-labelled proteomics, Label-free quantification, Autologous hematopoietic stem cell transplantation, Multiple myeloma

## Abstract

**Background:**

Oral mucositis is a frequently seen complication in the first weeks after hematopoietic stem cell transplantation recipients which can severely affects patients quality of life. In this study, a labelled and label-free proteomics approach were used to identify differences between the salivary proteomes of autologous hematopoietic stem cell transplantation (ASCT) recipients developing ulcerative oral mucositis (ULC-OM; WHO score ≥ 2) or not (NON-OM).

**Methods:**

In the TMT-labelled analysis we pooled saliva samples from 5 ULC-OM patients at each of 5 timepoints: baseline, 1, 2, 3 weeks and 3 months after ASCT and compared these with pooled samples from 5 NON-OM patients. For the label-free analysis we analyzed saliva samples from 9 ULC-OM and 10 NON-OM patients at 6 different timepoints (including 12 months after ASCT) with Data-Independent Acquisition (DIA). As spectral library, all samples were grouped (ULC-OM vs NON-OM) and analyzed with Data Dependent Analysis (DDA). PCA plots and a volcano plot were generated in RStudio and differently regulated proteins were analyzed using GO analysis with g:Profiler.

**Results:**

A different clustering of ULC-OM pools was found at baseline, weeks 2 and 3 after ASCT with TMT-labelled analysis. Using label-free analysis, week 1–3 samples clustered distinctly from the other timepoints. Unique and up-regulated proteins in the NON-OM group (DDA analysis) were involved in immune system-related processes, while those proteins in the ULC-OM group were intracellular proteins indicating cell lysis.

**Conclusions:**

The salivary proteome in ASCT recipients has a tissue protective or tissue-damage signature, that corresponded with the absence or presence of ulcerative oral mucositis, respectively.

**Trial registration:**

The study is registered in the national trial register (NTR5760; automatically added to the International Clinical Trial Registry Platform).

**Supplementary Information:**

The online version contains supplementary material available at 10.1186/s12903-023-03190-w.

## Background

Oral mucositis (OM) is an inflammatory condition of the oral mucosa caused by chemotherapy and/or radiotherapy. After high dose melphalan and autologous hematopoietic stem cell transplantation (ASCT), OM is seen in 90% of the patients, with severe forms in 46% of the patients [1]. It is clinically characterized by erythema, edema, and ulcerations and negatively affects patients’ quality of life. The development of ulcerations during the immunosuppressed state of ASCT recipients may enable bacteria to penetrate the bloodstream resulting in a systemic infection or even death [2, 3].

The pathobiology of OM as described by Sonis 15 years ago consists of 5 phases [4]. In the initiation phase, chemotherapy and/or radiotherapy cause damage to mucosal tissues, inducing the release of reactive oxygen species (ROS) and the activation of the transcription factor nuclear factor kappa-B (NF-κB). This leads to up-regulation of pro-inflammatory cytokines like tumor necrosis factor-α (TNF-α) and interleukins IL-1β and IL-6, which forms a positive feedback loop in the third phase. Ulcerations are developed in the fourth phase and products of bacterial cell walls can stimulate macrophages resulting in further release of pro-inflammatory cytokines and matrix metalloproteinases. In the final, healing phase, the migration, proliferation and differentiation of epithelial cells will renew the epithelium [4].

Multiple studies have focused on the prediction of (oral) mucositis. Next to mainly unmodifiable treatment related risk factors, genomic studies have found mutations associated with mucositis risk in genes involved in drug metabolism, cell growth, repair and inflammatory and immune pathways [5]. Proteomics analysis can indicate if those results at the genetic level are translated to protein expression level.

In a label-free quantification proteomics experiment operated in conventional data-dependent acquisition mode (DDA), the most abundant ion peaks are selected on the first mass spectrometry spectrum for fragmentation and subsequently identification. With the advances within the proteomics field, the sensitivity and reproducibility has increased with the development of the data independent acquisition mode (DIA) [6]. Using DIA, all detected precursor ions are fragmented within isolation windows covering the complete mass-to-charge range [6].

Whole-mouth saliva, a watery mixture of proteins derived from mainly salivary glands, but also blood, crevicular fluid and epithelial cells plays a major role in the protection of oral mucosa and teeth. Reductions in salivary flow rates and/or changes in salivary protein composition, hampers this protective function [7]. Changes in salivary function and protein composition have been reported after a HSCT. Salivary protein composition in the first three weeks after HSCT mainly reflected an inflammatory response, while salivary flow rates were decreased for several days or months [8].

Since saliva collection is easy and non-invasive, it is increasingly used as a diagnostic tool. Omics analyses including proteomics, have identified biomarkers of oral cancer and systemic cancers like gastric cancer in saliva [9, 10]. Using an ultra-deep proteomics approach 5,500 proteins, including intracellular and microbial proteins, were identified in saliva [11]. A proteomics study using unstimulated whole-mouth saliva from head and neck cancer patients undergoing radiotherapy found differences in the proteome of saliva collected before treatment that could correctly identify 90% of the patients that would develop OM after radiotherapy [12].

The aim of this study was to identify differences in the salivary proteome at different timepoints before, during and after HSCT between patients who developed ulcerative OM (ULC-OM) and those who did not (NON-OM). Two different proteomics approaches were adopted for proteomics analysis. Firstly, a pilot TMT-labelled proteomics analysis was performed. In order to detect lower abundant proteins, label-free quantification proteomics was then performed in DIA mode using a spectral library generated in DDA mode.

## Methods

A pilot TMT 10-plex experiment and a label-free (DDA/DIA) approach were used in this study. All saliva samples for both proteomics experiments were selected from multiple myeloma patients receiving autologous HSCT (ASCT) after high-dose melphalan (200 mg/m^2^) who were included in the multicenter, longitudinal H-OME study, a Dutch extension of the Orastem study [13]. The H-OME study (funded by Dutch Cancer Society, ACTA 2014–7468; trial register NTR5760) was approved by the Medical Research Ethical Committee (NL52117.018.15) and conducted according to GCP guidelines and the declaration of Helsinki. All patients signed informed consent before participation.

During the H-OME study, OM was scored 3 times a week during the hospitalization phase using the WHO scoring system. Patients with a score of ≥ 2 (indicating ulcerative OM) during this period were considered as ULC-OM patients [14]. Two patients were in complete remission (1 in the ULC-OM group and 1 in the NON-OM group), the other patients were in partial remission prior to ASCT.

Chewing stimulated whole-mouth saliva samples were collected at multiple time points: before ASCT during the focal dental evaluation (baseline; median of 40 days before ASCT (range: 14 – 124 days before ASCT)), once a week during the hospitalization phase for the ASCT and 3 and 12 months after ASCT, as previously described [15]. In short, patients were asked to refrain from eating and drinking for 1 h prior to collection. SWS was collected on ice for 2 – 5 min by chewing on a neutral chewing gum base, after swallowing and 1 min of chewing. Within 2 h of collection, saliva samples were centrifuged for 5 min at 9,800. The supernatant was separated from the pellet and stored at -80 °C.

The mass spectrometry proteomics data of both the TMT and label-free experiments have been deposited to the ProteomeXchange Consortium [16] via the PRIDE [17] partner repository with the dataset identifiers PXD033603 for the TMT experiment, PXD033591 for the DDA part and PXD033525 for the DIA part.

### TMT 10-plex experiment

**Fig. 1:**
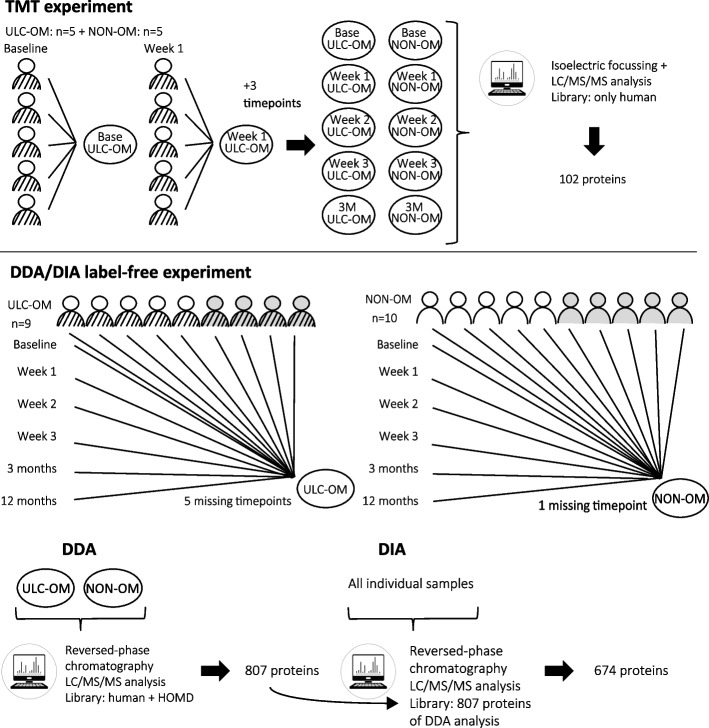
Schematic overview of the TMT-labelled, data dependent analysis (DDA) and data-independent analysis (DIA) experimental designs. TMT experiment: stimulated whole-mouth saliva samples were pooled from 5 ulcerative oral mucositis (ULC-OM) and 5 non-oral mucositis (NON-OM) patients at 5 different time points (baseline, 0–4 days (week 1), 6–11 days (week 2), 13–18 days (week 3) and 3 months). The resulting 10 pools were labelled and analyzed with LC/MS/MS. Label-free quantification (LFQ) experiment: for the DDA analysis, stimulated whole-mouth saliva samples from 9 ULC-OM and 10 NON-OM patients at 6 different time points (baseline, 0–4 days (week 1), 6–11 days (week 2), 13–18 days (week 3), 3 months and 12 months) were grouped resulting in 2 groups that were analyzed with LC/MS/MS. Of the ULC-OM patients, 5 saliva samples could not be collected, resulting in 5 missing timepoints (week 2, twice week 3 and twice 12 months). Only 1 saliva sample of a NON-OM patient at week 1 could not be collected. The oblique striped jackets represent ULC-OM patients and the empty jackets represent NON-OM patients. Patients selected for the TMT experiment were also used in the LFQ experiment in addition to 4 ULC-OM or 5 NON-OM other patients (in grey). For the DIA analysis, individual samples were analyzed with LC/MS/MS using the with DDA generated library

For further experimental details and settings see Additional file 1. Five ULC-OM and 5 NON-OM patients (WHO score of 0) (ULC-OM: median age 57 (33 – 63 year); NON-OM: median age 53 (52 – 56 years)) were selected for this experiment based on the availability of a large volume of saliva, and of all time points (excluding 12 months follow-up as these samples were not yet available). Equal volumes of stimulated whole-mouth saliva samples (11 μl) from 5 ULC-OM and 5 NON-OM patients were pooled for each time point, resulting in 10 pools (Fig. 1). Those pools were reduced, alkylated, digested with trypsin and subsequently labeled with a unique TMT 10-plex label (Thermo Fisher Scientific; for experimental details see Additional file 1). All uniquely labeled pools were combined into one and separated by isoelectric focusing. Fractions were combined (fraction 1 and 7, fractions 2 and 8 and so on) to yield 6 fractions for LC/MS/MS analysis. An extended 120 min gradient chromatographic separation (EASY NanoLCsystem; Thermo Fisher Scientific, UK) for each combined fraction was used. Electrospray ionization was used on an Orbitrap Velos Pro (Thermo Fisher Scientific, UK) and for peptide identification and reporter ion fragmentation, the top 10 precursor ions (intensity-based selected in data-dependent switching mode) were fragmented by Higher-energy C-trap dissociation (HCD).

#### Protein identification and quantification

Raw data files were converted to mgf files using MSConvertGUI of ProteoWizard using peak picking filtering by the Vendor algorithm and default settings [18]. For peptide and protein identification, mgf files were searched against the Homo Sapiens Swiss-Prot database (September 2018 containing 20,362 entries, pig trypsin (P00761) was added) with the MS-GF + (v2018.04.09) and OMSSA algorithms in SearchGUI 3.3.4 and PeptideShaker 1.16.29 [19–22]. The validated proteins at a 1% false-positive rate were further analyzed with Reporter 0.7.20 using default settings to obtain quantitative results. From the output, the ratios were used for data analysis. For further details and settings see Additional file 1.

#### Data analysis

To explore patterns between the ULC-OM and NON-OM pools and the different time points, a principal component analyses (PCA) and a heatmap were generated using RStudio (version 1.2.5001) on log_2_ transformed ratios from Reporter. Distance calculation for the heatmap was based on a correlation measurement as described in equation 3 in Key (2012) [23].

Per timepoint, fold changes of ratios for ULC-OM and NON-OM pools were calculated and log_2_ transformed. Z-scores were calculated and a score of ≤ -2.0 or ≥ 2.0 was used as a threshold to identify proteins that were either up- or down-regulated in the ULC-OM pool at a specific time point. Those proteins were further analyzed using Reactome [24] in a pathway analysis. Uniprot was used for proteins that were not identified in Reactome [25].

#### Label-free quantification (LFQ) experiment using data dependent and data independent analysis (DDA/DIA)

In total 9 ULC-OM and 10 NON-OM patients (ULC-OM: median age 57 (33 – 69 year), 6 males; NON-OM: median age 57 (52 – 66 years), 6 males) were selected for this experiment (Fig. 1). The same patients as used in the TMT experiment were included with 4 (ULC-OM) or 5 (NON-OM) additional patients, also selected based on sample time points and volume of saliva available. For all stimulated whole-mouth saliva samples, total protein concentration was determined by absorbance 280 nm (Nanodrop spectrophotometer). To generate a spectral library for DIA analysis, 10 μg of total protein from each sample was used to create a group consisting of all saliva samples for ULC-OM or NON-OM patients and all time points. 100 μg of the ULC-OM and NON-OM group was added to solid urea. Proteins were subsequently reduced, alkylated and digested with lysyl endopeptidase and trypsin (for experimental details see Additional file 1). This peptide mixture was purified and fractionated using reversed-phase chromatography at pH 5.5 using an Agilent 1100 series HPLC. Fractions were collected in MS vials per minute over a time interval of 65 min and automatically pooled by restarting the fraction collection cycle every 10 min resulting in 10 pooled fractions. Fractions were further analyzed in an LC/MS/MS analysis on an Ultimate 3000 RSLC nano-LC (Thermo) in line connected to a Q Exative HF mass spectrometer (Thermo). Trapping was performed at 10 μl/min for 4 min in loading solvent A on a 20 mm trapping column (made in-house, 100 μm internal diameter (I.D.), 5 μm beads, C18 Reprosil-HD, Dr. Maisch, Germany). Peptides were separated on a 200 cm μPAC™ column with C18-endcapped functionality (Thermo, Belgium) kept at a constant temperature of 50 °C. Peptides were eluted by a staggered gradient reaching 33% MS solvent B (0.1% FA in water/acetonitrile (2:8, v/v)) in 105 min, 55% MS solvent B in 145 min and 99% MS solvent B in 150 min at a constant flow rate of 300 nl/min, followed by a 10-min wash at 99% MS solvent B and re-equilibration with MS solvent A (0.1% FA in water). In the first 15 min the flow was set to 750 nl/min. The mass spectrometer was operated in data-dependent mode, automatically switching between MS and MS/MS acquisition for the 8 most abundant ion peaks per MS spectrum. Full-scan MS spectra (375–1500 m/z) were acquired at a resolution of 60,000 in the Orbitrap analyzer after accumulation to a target value of 3,000,000 at a maximum fill time of 60 ms. The 8 most intense ions above a threshold value of 8300 were isolated for fragmentation at a normalized collision energy of 28% after filling the trap at a target value of 100,000 for maximum 120 ms. MS/MS spectra (200–2000 m/z) were acquired at a resolution of 15,000 in the Orbitrap analyzer.

### Protein identification and quantification DDA

All LC/MS/MS runs were searched together using the Andromeda search engine within MaxQuant version 1.5.6.5. Default settings including a false discovery rate of 1% at the peptide and protein level were used to search the spectra against the Homo Sapiens Swiss-Prot database (January 2019 containing 20,413 human protein sequences) and the Human oral Microbiome Database (HOMD; www.HOMD.org). Proteins were quantified by the MaxLFQ algorithm integrated into the MaxQuant software (PMID 24942700). Only proteins with at least one unique or razor peptide were retained for identification, while a minimum ratio count of two unique peptides was required for quantification. Protein identifications through mixing of microbial peptides with human peptides was avoided using the split taxonomy feature in the MaxQuant search engine in accordance to Grassl et al*.* 2016 [11].

### Data analysis DDA

Log_2_ transformed LFQ intensities were used to calculate NON-OM/ULC-OM fold change for each protein. The standard deviation of the median subtracted distribution was used to determine up- and down-regulated proteins according to upper and lower limit of the 95% confidence interval. The up- and down-regulated proteins were added to the unique proteins and used in a gene ontology (GO) analysis using g:Profiler [26]. The unique and up-regulated proteins in the ULC-OM and NON-OM groups were run as multi query. Only annotated genes were used, and Bonferroni correction was used for the significance threshold. The significant GO terms for biological processes (BP) and cellular components (CC) were further analyzed using RStudio (version 1.1.463) and parent terms were used to divide BP and CC terms into 4 and 3 groups, respectively. The BP terms were divided into 4 subgroups: immune (BP terms only under the immune system process (GO:0002376)), localization (BP terms only under localization (GO:0051179)), both terms, and others. The CC terms were divided into 3 subgroups: extracellular proteins (GO:0005576), intracellular proteins (GO:0005622) and, others. To determine if the ULC-OM and NON-OM groups were similar in significance for GO terms within specific subgroups, paired T-tests were used on -log10 transformed adjusted *p*-values of each GO term of both groups.

### DIA analysis

Half of the individual patient samples collected at different timepoints from 19 patients suffering from ulcerative OM or not, used for DDA analysis, were re-dissolved in 20 μl of which 5 μl was used for LC/MS/MS Data Independent Acquisition (DIA) analysis. The LC conditions were kept equal to the DDA analysis. DIA settings were set at an isolation window of 10 m/z with overlapping windows in a m/z range of 400–900 m/z. The resolution for MS2 was set to 15,000 with a collection of 3,000,000 ions with a maximum fill time of 45 ms and a normalized collision energy for fragmentation of 30. After every set of 30 MS2 windows, an MS1 was recorded with the same settings as in the DDA analysis, except for the maximum fill time now allowing for only 50 ms and a scan range of 200–2000 m/z. The dataset was analyzed with the Spectronaut software (v13.8) using the spectral library from the DDA analysis. Default parameters were used, except for the proteotypicity of the peptides, only unique was selected. The signals were normalized across all runs and pairwise t-tests were performed to compare ULC-OM versus NON-OM across all timepoints and per timepoint. To reveal proteins with a significantly different expression level between different conditions and timepoints, two-way ANOVA was performed to compare intensities of proteins in the condition state (ULC-OM vs NON-OM) with the timepoint group. Differently regulated proteins were analyzed using GO analysis with g:Profiler, with similar settings as the DDA results, except that the lists were run as single query. The PCA plot and volcano plot were generated in Rstudio (version 1.1.463; ggplot2 library). Log2 fold changes were calculated per protein to compare week 1, 2 and 3 intensities (the hospitalization period) with intensities outside this period (baseline, 3 months and 12 months). *P*-values of the volcano plot were calculated using multilevel linear regression analysis per protein (a minimal of 3 intensities had to be known per protein per period (within or outside the hospitalization period) to calculate the *p*-value. *P*-values were corrected using the ‘FDR’ function of the p.adjust package.

## Results

### TMT 10-plex experiment

**Fig. 2:**
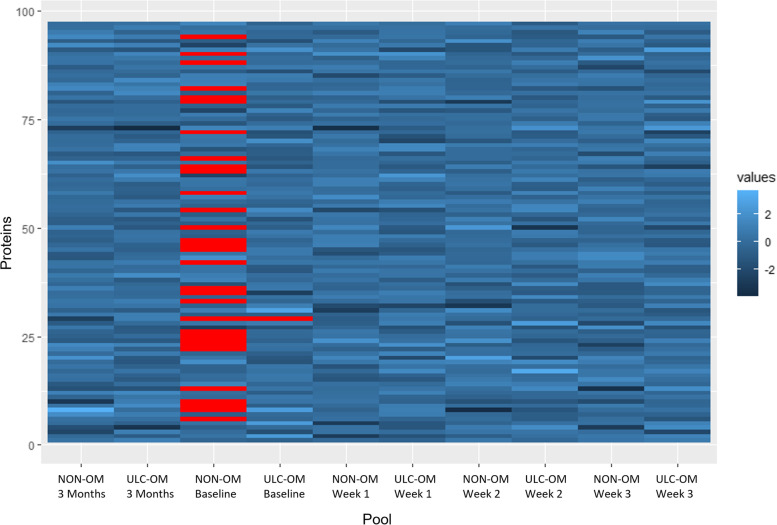
Missing values (red) of the Log_2_ transformed ratios (blue) of the different pools in the TMT-labelled experiment

**Fig. 3:**
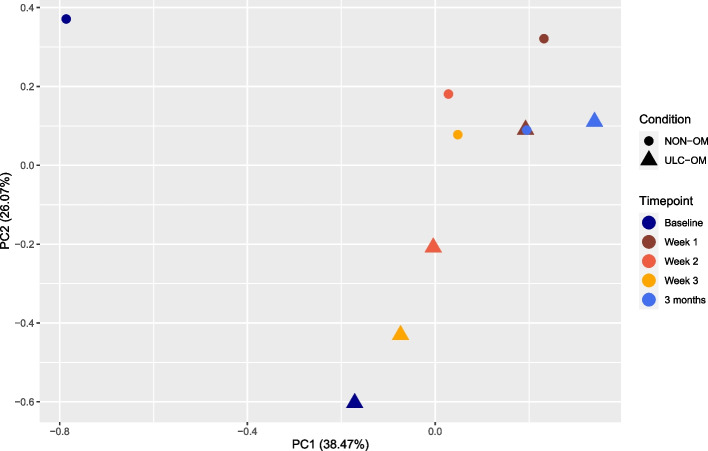
Principal component analysis (PCA) plot of the TMT-labelled experiment with colors indicating the different timepoints (blue colors: baseline and 3 months; orange colors: weeks 1–3 (hospitalization)) and shapes indicating the condition: ulcerative oral mucositis (ULC-OM, filled round) or non-oral mucositis (NON-OM, filled triangle)

**Fig. 4:**
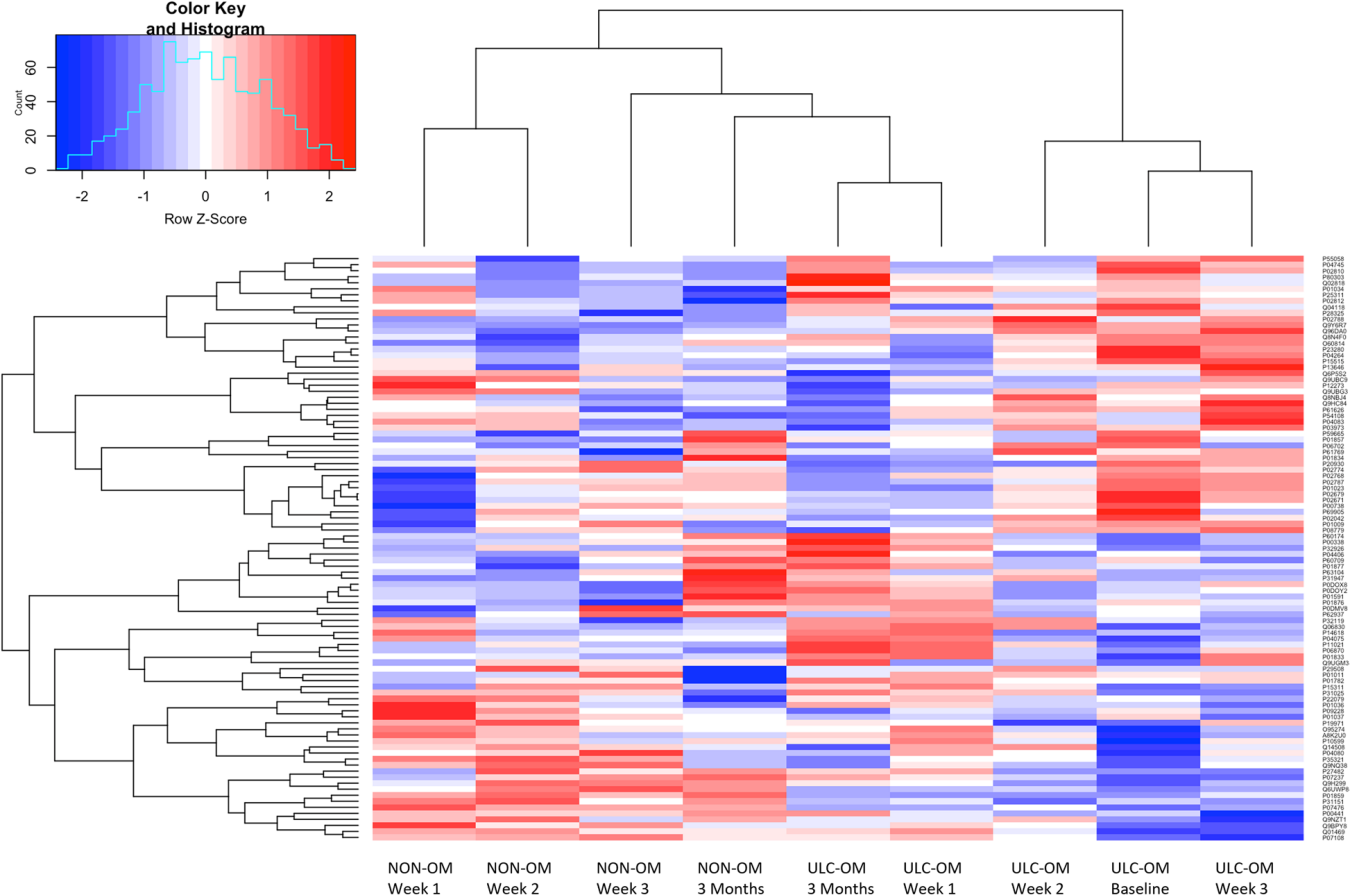
Heatmap of the TMT experiment with the different timepoints (Baseline, Week 1, Week 2, Week 3 and 3 Months) for the ulcerative oral mucositis (ULC-OM) and non-oral mucositis (NON-OM) pools. The baseline NON-OM pool is excluded, since it is an outlier in the PCA plot (Fig. 3)

In the TMT experiment 102 validated proteins were identified of which 97 proteins could be quantified. Of the 97 quantified proteins, 31 proteins could not be quantified in all pools resulting in missing values, especially the baseline NON-OM pool (Fig. 2). To explore patterns for all proteins between ULC-OM and NON-OM pools at different time points, a principal component analysis (PCA) was performed (Fig. 3). Based on 2 components, ULC-OM and NON-OM pools could be separated and the main component could separate baseline, week 2 and week 3 ULC-OM pools as a cluster. Also in the heatmap a similar distinct clustering of the baseline, week 2 and week 3 ULC-OM pools is seen, although there are no clear clusters of differently correlated proteins (Fig. 4). Since less proteins could be quantified for the baseline NON-OM pool and this sample clustered different in the PCA plot compared with the other samples, the baseline NON-OM pool is considered as an outlier and excluded in the heatmap (Fig. 4).

Fold changes between ULC-OM and NON-OM pools to determine up-regulated and down-regulated proteins in the ULC-OM pool were calculated per time point and are listed in Supplementary Table 1A, Additional file 2. Across all time points, in the ULC-OM pools 15 different proteins were up-regulated and 7 different proteins were down-regulated versus the NON-OM pools. The involved pathways in Reactome and/or function of up- or down-regulated proteins according to Uniprot are listed in Supplementary Table 1B, Additional file 2 per time point. At baseline and 6–11 days after ASCT proteins involved in gene expression and transcription were up-regulated and at 13–18 days proteins involved in immune system pathways were up-regulated. Down-regulation of proteins involved in innate immune system pathways occurred at 0–4 days, 6–11 days and 3 months after ASCT. The protein involved in ‘mitochondrial fatty acid beta-oxidation’ was downregulated at 13–18 days after ASCT.

### Data Dependent analysis (DDA)

**Fig. 5:**
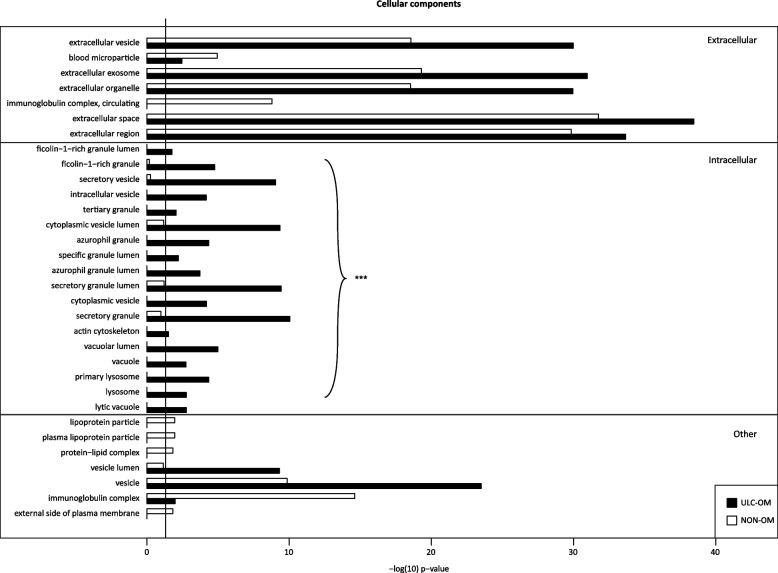
Bar graph of significant *cellular components* gene ontology (GO) terms for unique and up-regulated proteins in the ulcerative oral mucositis (ULC-OM; black bars) and non-oral mucositis (NON-OM; white bars) groups of the label-free quantification Data Dependent Analysis (DDA) experiment. The vertical line represents significance threshold of *p* = 0.05. The intracellular CC GO terms were more significant in the ULC-OM group compared to the NON-OM group (ULC-OM: (mean ± SD) 10.77 ± 6.54, NON-OM: 0.48 ± 0.98, *p* < 0.001, 95% CI [7.44, 13.13] (shown with accolade and ***)

**Fig. 6:**
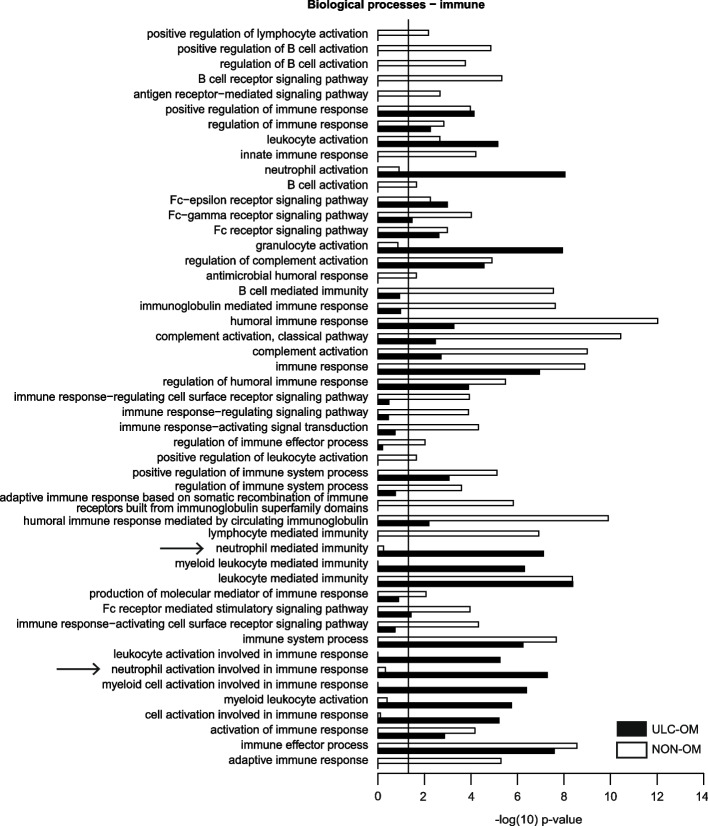
Bar graphs of significant gene ontology (GO) terms related to subgroup *immune* within *biological processes* for unique and up-regulated proteins in the ulcerative oral mucositis (ULC-OM; black bars) and non-oral mucositis (NON-OM; white bars) groups of the label-free quantification Data Dependent Analysis (DDA) experiment. The vertical line represents significance threshold of *p* = 0.05. Neutrophil degranulation and activation terms (mentioned in the Discussion section) are indicated with an arrow

**Fig. 7:**
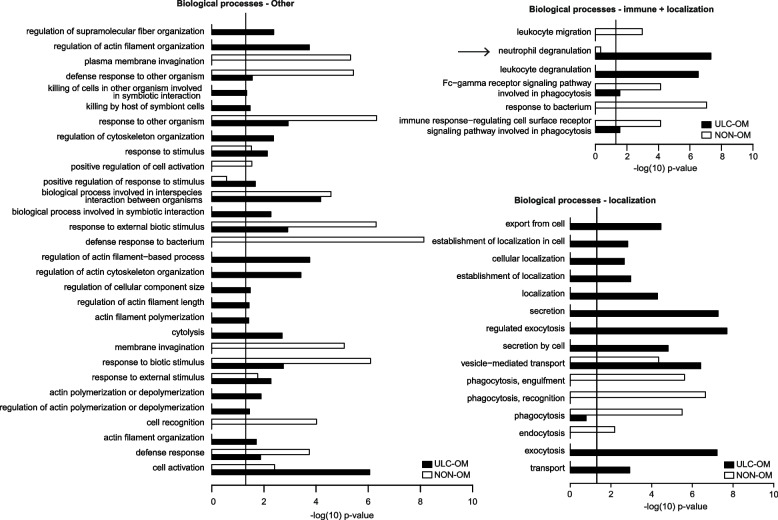
Bar graphs of significant gene ontology (GO) terms related to subgroups *other, immune* + *localization and localization* within the *biological processes* for unique and up-regulated proteins in the ulcerative oral mucositis (ULC-OM; black bars) and non-oral mucositis (NON-OM; white bars) groups of the label-free quantification Data Dependent Analysis (DDA) experiment. The vertical line represents significance threshold of *p* = 0.05. Neutrophil degranulation and activation terms (mentioned in the Discussion section) are indicated with an arrow

To generate a spectral library for DIA analysis, all saliva samples at different timepoints of ULC-OM and NON-OM patients were grouped resulting in an ULC-OM and a NON-OM group (Fig. 1). In both groups a total of 696 human and 111 microbial proteins were identified of which 693 human and 106 microbial proteins were quantified. Of those 799 quantified proteins, 96 human and 42 microbial proteins could only be quantified in ULC-OM group and 86 human and 39 microbial proteins could only be quantified in the NON-OM group. Among the 511 human proteins quantified in both groups, 10 proteins were significantly up-regulated in the ULC-OM group and 13 proteins were significantly up-regulated in the NON-OM group. Gene ontology analysis for cellular components and biological processes among the unique and up-regulated proteins are shown in Figs. 5, 6 and 7. The intracellular CC GO terms were more significant in the ULC-OM group compared to the NON-OM group (ULC-OM: (mean ± SD) 10.77 ± 6.54, NON-OM: 0.48 ± 0.98, *p* < 0.001, 95% CI [7.44, 13.13]; Fig. 5). Within subgroups of the biological processes, GO terms in the immune subgroup were more significant in the NON-OM group compared to the ULC-OM group (ULC-OM 6.56 ± 6.41, NON-OM 9.64 ± 7.03, *p* = 0.036, 95% CI [-5.97, -0.21]; Figs. 6 and 7).

**Fig. 8:**
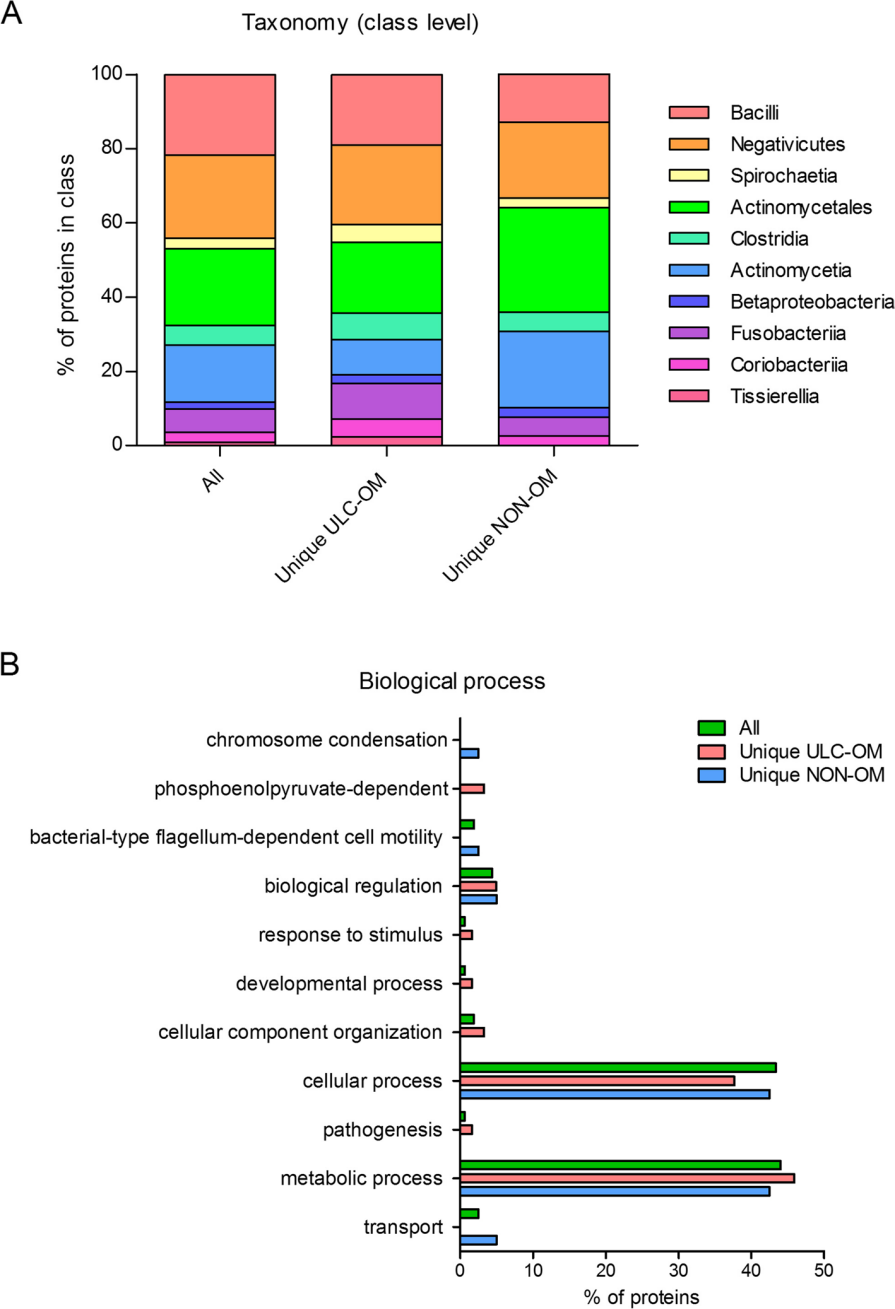
Taxonomy at class level (**A**) of microbial proteins and biological processes of those proteins (**B**). ‘All’ represents all identified microbial proteins, in ‘unique ULC-OM’ or ‘unique NON-OM’ only the uniquely identified proteins in the ulcerative oral mucositis (ULC-OM) or non-oral mucositis (NON-OM) group are shown (Data Dependent Analysis (DDA) experiment)

Of the total number of identified proteins, 13.7% were microbial. Among these, 1 protein from *streptococcus* (TfoX_N domain-containing protein) covered 82% of the whole microbial proteome present. Compared to the total salivary proteome, this is only 1%. Gene ontology analysis among the microbial proteins revealed that the proteins were mainly derived from *bacilli, negativicutes* and *actinomycetales* classes and were mainly involved in metabolic and cellular processes with only small differences between unique microbial proteins of the ULC-OM and NON-OM groups (Fig. 8).

**Fig. 9:**
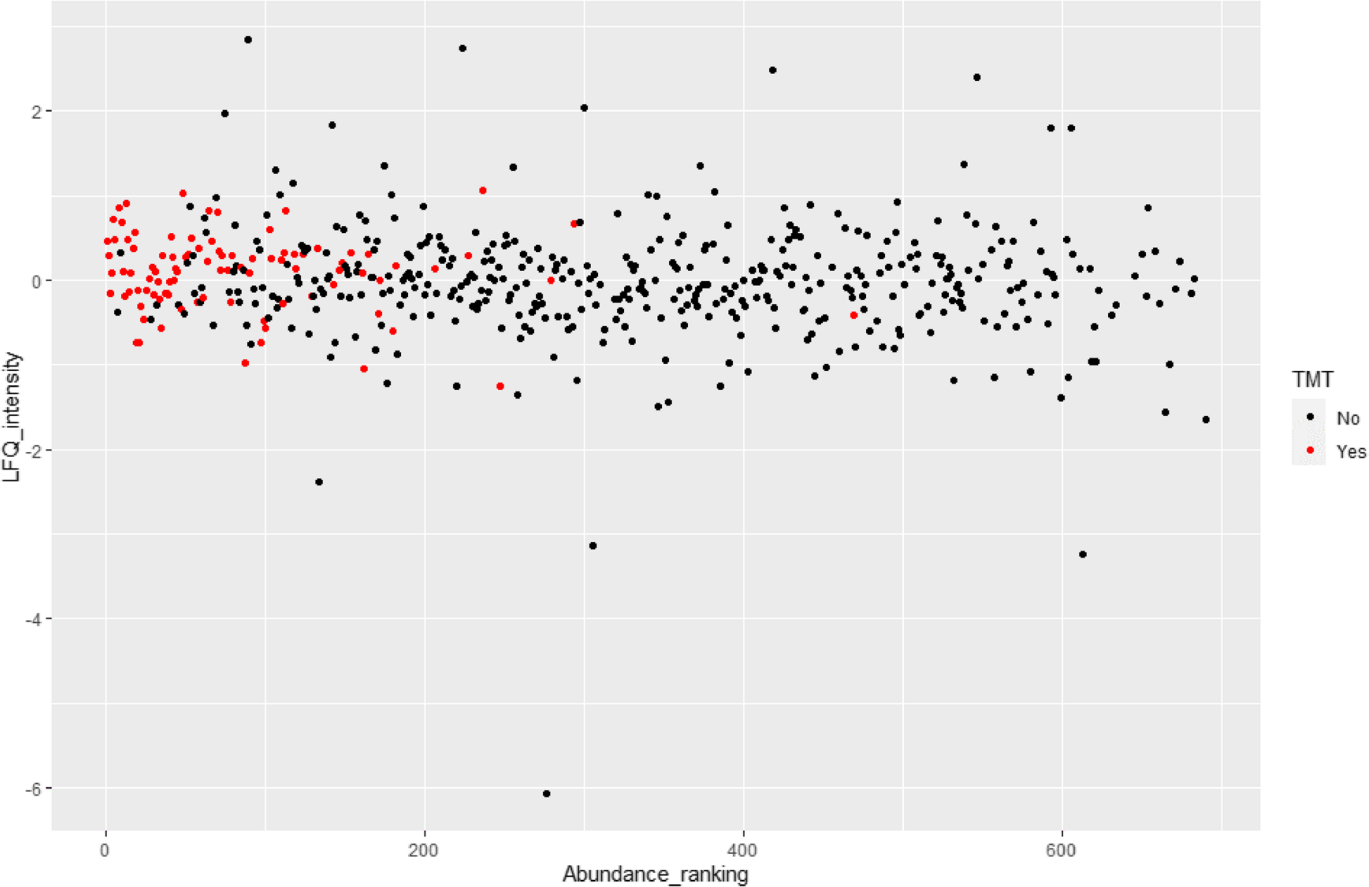
Dot plot of the log_2_ transformed label-free quantification intensity (LFQ) and corresponding abundance ranking of the identified proteins in the DDA experiment. The red dots are the proteins identified in both the DDA and TMT-labelled experiment

With the label-free quantification method, more proteins and especially more low abundant proteins were identified compared to the TMT-labelled experiment (Fig. 9). Five proteins identified in the TMT-labelled experiment were not identified in the DDA analysis or were considered as a contaminant: serum albumin, filaggrin, immunoglobulin heavy constant alpha 2, keratin type 1 cytoskeletal 13, keratin type 1 cytoskeletal 16. Only 2 proteins from the uniquely identified and up-regulated proteins in the DDA analysis were identified in the TMT-labelled analysis: histatin 1 and small proline-rich protein 2A.

### Data Independent Acquisition (DIA)

**Fig. 10:**
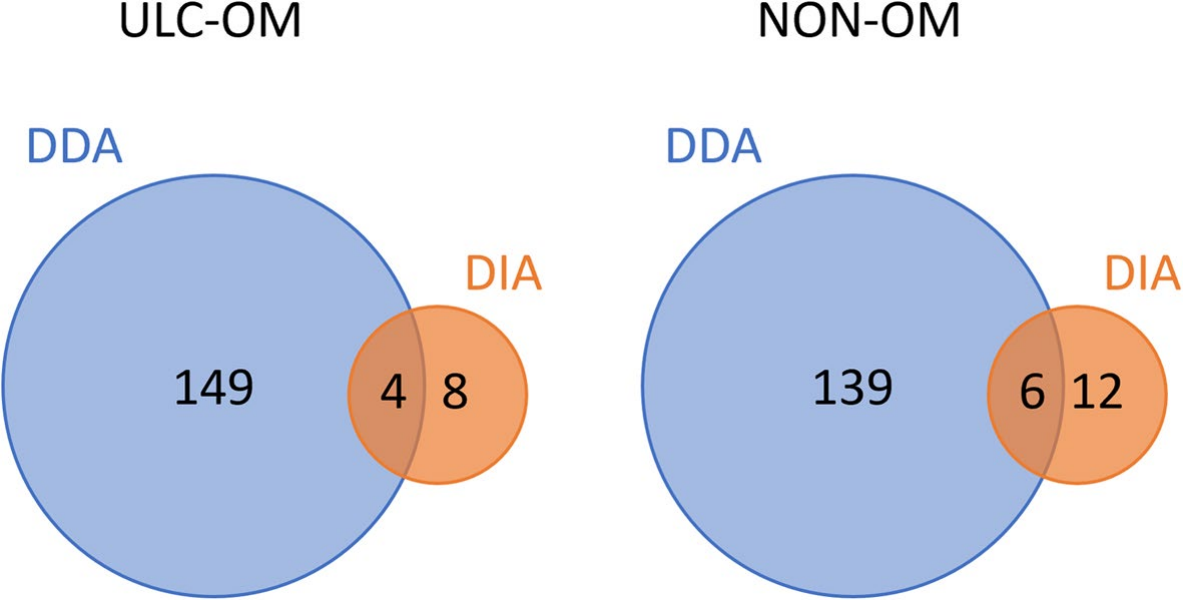
Venn diagrams showing the overlap of the regulated proteins between Data Dependent Analysis (DDA) and Data Independent Analysis (DIA) of the ulcerative oral mucositis (ULC-OM) and non-oral mucositis (NON-OM) samples

To detect lower abundant proteins, Data Independent Acquisition (DIA) was used to analyse 103 stimulated whole-mouth saliva samples collected at different timepoints from 19 different patients suffering from ulcerative OM or not. In this DIA analysis, 674 proteins (647 human and 27 microbial proteins) were identified and quantified. Pairwise comparison between ULC-OM and NON-OM over all timepoints in a single t-test with multiple sample correction (FDR = 0.05 and S0 = 1) resulted in 20 significantly up-regulated proteins (Table 1). Although some overlap is found between the up-regulated proteins in the DDA and DIA analysis, the number of up-regulated proteins is much smaller in the DIA analysis (Fig. 10). The 12 significantly up-regulated proteins in the NON-OM group were significantly enriched in mainly immune response related biological processes, while no significantly enriched biological processes were found for the up-regulated proteins in the ULC-OM patients (Supplementary Table 2, Additional file 3). A search in the Reactome database revealed that those proteins were mainly related to metabolism, developmental biology and signal transduction. At the different timepoints, no significantly different proteins could be identified, except for keratin type II cytoskeletal 6B which was significantly upregulated in ULC-OM patients at week 3.

**Table 1:** Significantly up-regulated proteins of pairwise comparison between ulcerative oral mucositis (ULC-OM) and non-oral mucositis (NON-OM) patients over all timepoints (Data Independent Acquisition (DIA) experiment)

Protein accession number	Protein name	Difference Log_2_ (ULC-OM/NON-OM)	-log *P*-value
**ULC-OM (*n* = 8)**			
O75116	Rho-associated protein kinase 2	2.228	4.348
Q8NCL4	Polypeptide N-acetylgalactosaminyltransferase 6	2.141	3.658
P32320	Cytidine deaminase	1.640	3.245
Q04118	Basic salivary proline-rich protein 3	1.552	2.425
Q6P4A8	Phospholipase B-like 1	1.121	4.263
Q8N1N4	Keratin, type II cytoskeletal 78	1.038	3.020
O15145	Actin-related protein 2/3 complex subunit 3	0.941	3.339
Q09666	Neuroblast differentiation-associated protein	0.844	3.928
**NON-OM (*n* = 12)**			
A0A075B6K4	Immunoglobulin lambda variable 3–10	-1.743	6.429
Q14602	Putative DNA-binding protein inhibitor ID-2B	-1.375	3.250
P01780	Immunoglobulin heavy variable 3–7	-1.348	3.867
P61916	NPC intracellular cholesterol transporter 2	-1.301	3.360
Q14116	Interleukin-18	-1.088	3.598
A0A0J9YX35	Immunoglobulin heavy variable 3-64D	-1.051	3.125
P54802	Alpha-N-acetylglucosaminidase	-1.038	3.553
A0A0C4DH68	Immunoglobulin kappa variable 2–24	-0.965	2.829
P01037	Cystatin-SN	-0.901	4.042
Q9BRF8	Serine/threonine-protein phosphatase CPPED1	-0.872	3.483
Q6P5S2	Protein LEG1 homolog	-0.846	3.341
P53634	Dipeptidyl peptidase 1 (Cathepsin C)	-0.715	6.348

Two-way ANOVA resulted in 34 significantly different expressed proteins (32 human and 2 microbial) between different conditions (Supplementary Table 3, Additional file 3). Human proteins were involved in metabolic and cellular processes including small molecule metabolic process and cornification. The two significantly different expressed microbial proteins were derived from *Streptococcus vestibularis and Atopobium species* and were involved in carbohydrate metabolism (degradation of carbohydrates and galactose metabolism). A heatmap after non-supervised hierarchical clustering revealed no clear clustering of significantly differently expressed proteins.

**Fig. 11:**
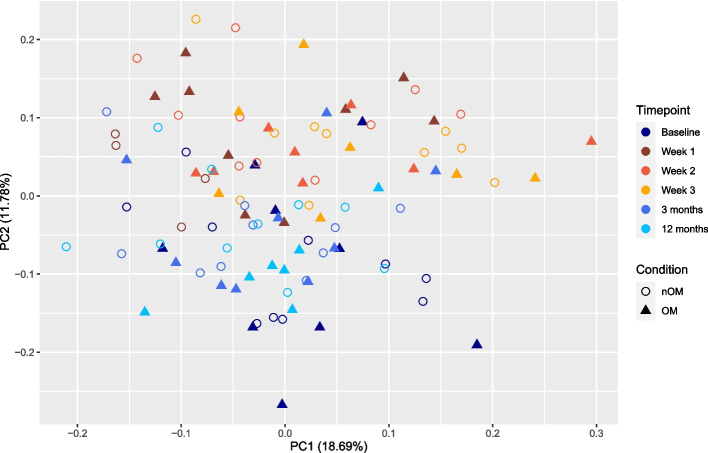
Principal component analysis (PCA) plot of the Data Independent Acquisition (DIA) experiment with colors indicating the different timepoints (blue colors: baseline, 3 and 12 months; orange colors: week 1–3 (hospitalization)) and shapes indicating the condition: ulcerative oral mucositis (ULC-OM, open round) or non-oral mucositis (NON-OM, filled triangle)

**Fig. 12:**
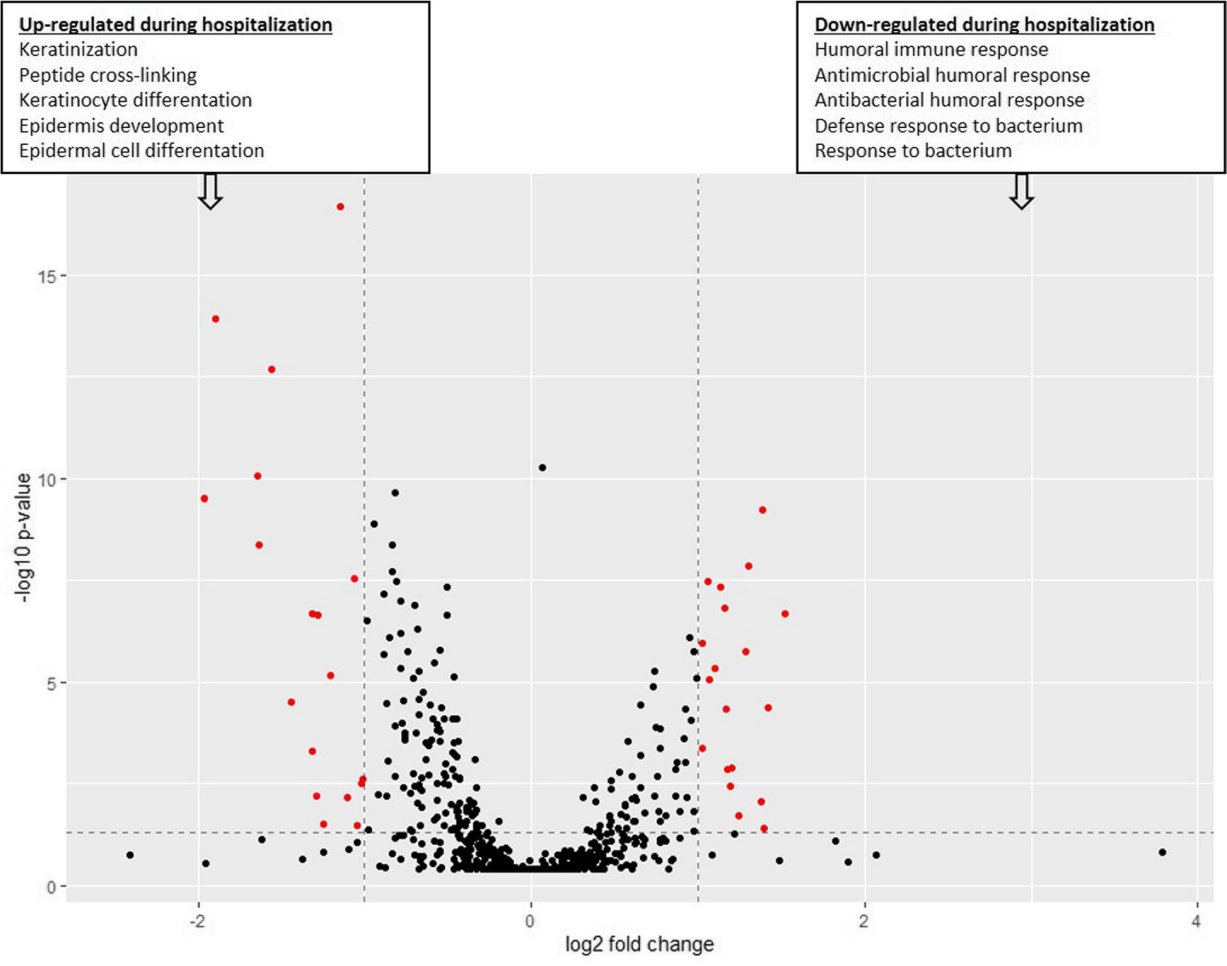
Volcano plot of differently regulated proteins (red dots) during week 1, 2 and 3 (hospitalization) or at baseline, 3 months and 12 months of the Data Independent Acquisition (DIA) experiment. The horizontal line indicates threshold for statistical significance (*p* < 0.05) and the vertical lines indicates threshold for differently regulated proteins (fold change of 2). In textboxes the top 5 of significantly enriched biological processes gene ontology terms are listed for upregulated proteins during hospitalization (left box) and outside the hospitalization period (right box). Differently expressed proteins and details of the gene ontology terms are listed in Supplementary Tables 4 and 5, Additional file 3

A PCA analysis could not differentiate clusters between ULC-OM and NON-OM samples, but some clustering is detected between different timepoints. Week 1–3 samples (during hospitalization) cluster more or less together, whilst baseline samples cluster with follow-up samples at 3 and 12 months after ASCT (Fig. 11). A comparison of the LFQ intensities between the hospitalization period and other timepoints revealed that 37 proteins were significantly differently regulated (Fig. 12; Supplementary Table 4, Additional file 3). During hospitalization, up-regulated proteins were mainly involved in keratinization, while down-regulated proteins during hospitalization (up-regulated at baseline/3 and 12 months) were mainly involved in antibacterial humoral immune response (Fig. 12; Supplementary Table 5, Additional file 3).

## Discussion

Two different proteomics techniques were used to compare the salivary proteome at different timepoints before, during and after ASCT of patients who developed ulcerative OM and those who did not. In total, 102, 807 and 674 proteins were identified, respectively, for the TMT-labelled experiment, DDA and DIA analysis of the label-free experiment. The PCA analysis of the DIA experiment showed a different clustering of samples over time, while there were no differences between ULC-OM and NON-OM samples. The 12 up-regulated proteins over all timepoints in NON-OM patients of the DIA experiment were significantly involved in mainly immune system related biological processes. Among the 807 proteins identified in the DDA experiment, 13.7% of the proteins were derived from the oral microbiome. Although no differences were observed in those microbial proteins, the unique and up-regulated human proteins of the NON-OM group were more involved in immune system related processes and those proteins in the ULC-OM group were more intracellular proteins.

Compared to the LFQ experiment, only few proteins were identified in the TMT-labelled experiment. The number of proteins identified in other proteomics studies in saliva also varies. More than 1400 proteins are identified in some studies [27], while around 500 proteins were identified in another TMT-labelled experiment in saliva samples after amylase depletion [10]. A labelled and a label-free proteomics approach of pooled saliva samples, identified 249 and 180 proteins, respectively [28, 29]. Comparing a 10-plex TMT experiment with a 6-plex TMT experiment and a 4-plex iTRAQ (isobaric tags for relative and absolute quantification) also identified fewer proteins in the 10-plex TMT experiment [30, 31]. Likely the labelling, the combination of fractions and the lack of depletion of high abundant proteins in our TMT-labelled experiment resulted in the low amounts of identified proteins. The use of a label-free DIA experiment clearly increased the number of identified proteins and increased the identification and quantification of low abundant proteins in our study.

In addition to the low number of proteins identified in the TMT-labeled experiment, one sample resulted in multiple missing values by quantification. The reason for those missing values is unknown. Although the pooling of samples in this experiment was performed based on equal volumes, the protein concentration did not differ much between the samples that were pooled in this experiment. Furthermore, no isotope correction during the quantification could be performed, which resulted in less accurate quantification. To determine the impact of this uncertainty, Reporter was run twice, once with default settings and once with all isotope corrections set to zero. This resulted in minor differences, only for the baseline NON-OM pool a larger difference was found. Therefore, the baseline NON-OM pool of the TMT-labelled experiment should be viewed with caution.

In this TMT experiment, up-regulated proteins in the ULC-OM pool were involved in gene expression at 6–11 days after ASCT, which is in accordance with the timing of the steps in the pathobiology model of oral mucositis. Especially in the first 3 phases, the activation of the transcription factor NF-κB leads to gene expression for the production of pro-inflammatory cytokines [4]. Those up-regulated proteins were also involved in DNA repair and cell cycle which supports the finding that genes related to DNA damage and cell cycle were associated with OM [32]. With this proteomics experiment, these genetic differences could be translated to the protein level. In a recent study, a decrease in salivary levels of the protein zymogen granule 16 homolog B, that was up-regulated at 6–11 days in ULC-OM compared to NON-OM, might suggest damage and dysfunction of the salivary glands [33].

The distinct clustering of samples during the hospitalization period compared to timepoints outside the hospitalization period (baseline, 3 and 12 months follow-up) support our earlier findings on salivary protein levels in the same study population [15]. In that study, total IgA and neutrophil defensin levels were significantly decreased in the second and third week of hospitalization [15]. This is in line with the finding that down-regulated proteins during hospitalization were involved in antibacterial immune response. Likely reflecting the effects of high-dose melphalan on the IgA producing plasma cells and neutropenia. This distinct clustering further coincides with the shift and less diverse oral microbiome in ulcerative oral mucositis patients as earlier reported in the same study population [34].

Although we found no differences between ULC-OM and NON-OM patients in salivary levels of earlier investigated antimicrobial proteins [15], several proteins were significantly different between ULC-OM and NON-OM patients using the grouped samples in the DDA experiment and over all timepoints with individual samples (DIA experiment). In the NON-OM samples and group, the unique and up-regulated proteins were mainly involved in biological processes related to B-cell activation and the adaptive immune response. This might suggest a more pronounced antibacterial immune response in NON-OM patients. IgA is the main immunoglobulin in saliva and is important for oral mucosal immunity by agglutination of microbes and inhibition of the adhesion of microbes to mucosal and dental surfaces [35]. The proteins related to the significantly enriched GO term for the NON-OM group ‘response to bacterium’ are mainly parts of immunoglobulins, which might suggest a higher concentration of immunoglobulins in saliva of NON-OM patients. Although we did not find significant differences in the total IgA concentrations in unstimulated and stimulated whole-mouth saliva between patients who developed ULC-OM and those who did not, the mean total IgA concentrations were slightly higher at most time points in the NON-OM patients [15].

While the B-cell immunity and bacterial defense GO terms were only significantly enriched among unique and upregulated proteins in the NON-OM group, the GO terms involved in neutrophil activation or neutrophil degranulation were more significantly enriched in those proteins of the ULC-OM group (DDA experiment, Figs. 6 and 7). Neutrophils play a key role in the innate immunity and defense against pathogens, but excessive activation of neutrophils can result in damage and exacerbate the tissue damage [36]. The activation and degranulation of neutrophils might be involved in the second and third phase of the pathobiology model of OM as described by Sonis, by the stimulation of macrophages and the activation of the primary damage response [4]. Although the results of our study suggests enhanced tissue damage, the neutrophils might be involved in the fifth phase in the epithelial repair as well [37]. One of the proteins underlying the neutrophil degranulation GO term is neutrophil elastase. This uniquely identified protein in the ULC-OM samples in this study is found in primary granules of neutrophils and is secreted at the end of degranulation together with other most pro-inflammatory and antimicrobial proteins [36, 38]. Also, in patients developing OM after radiotherapy for head and neck cancer this protein was significantly up-regulated before treatment [12]. Significant up-regulation of keratin type II cytoskeletal 6B in ULC-OM patients at week 3 (DIA analysis) also suggests additional damage compared to NON-OM patients. Together with keratin 16 and 17, keratin 6 is involved in the regeneration and migration of epidermal keratinocytes and is inducible upon injury and inflammation [39, 40].Taken together these results suggest a better or more pronounced bacterial defense in NON-OM patients while in ULC-OM patients the presence of activated neutrophils are associated with enhanced tissue damage. This supports the use of innate immune inhibitors as new therapy targets for OM, like dusquetide that enhances clearance of bacterial infection and dampens inflammation [41, 42]. The antibacterial immune response in NON-OM patients probably results in a more balanced interplay between the oral microbiome and the oral proteome, since the oral microbiome of NON-OM patients was more resilient compared to OM patients [34]. Further research might clarify the host-microbiome interactions in OM by linking the data obtained from the oral microbiome and the salivary proteome.

## Conclusions

Using labelled and label-free proteomics techniques, differences in the salivary proteome were found between ULC-OM and NON-OM patients. While DIA analysis mainly found differences in the hospitalization phase versus baseline and 3 and 12 months after ASCT, grouped samples and comparison between ULC-OM and NON-OM independent of time suggested that the salivary proteome of NON-OM patients has a tissue-protective signature compared to the salivary proteome of ULC-OM patients that has a damage signature.

## Supplementary Information

**Additional file 1. **Experimental details of the TMT-labelled and Label-Free Quantificationexperiment.

**Additional file 2: Supplementary Table 1A and 1B.** Listing the up- and down-regulated proteins in the ULC-OM pools versus the NON-OM pools and the involved pathways of these up- and down-regulated proteinsof the TMT-labelled experiment.

**Additional file 3: Supplementary Tables, 2, 3, 4 and 5** of the DIA experiment. Listing the significant enriched biological processes GO-terms of the significantly regulated proteins in the NON-OM samples across all timepoints; the significantly expressed proteins of the two-way ANOVA of the DIA experiment; the differently expressed proteins during the hospitalization period or outside the hospitalization period); and the top 5 of the significantly enriched biological processes GO-terms of those proteins listed in Supplementary Table 4.

## Data Availability

The mass spectrometry proteomics data of both the TMT and label-free experiments have been deposited to the ProteomeXchange Consortium [16] via the PRIDE [17] partner repository with the dataset identifiers PXD033603 for the TMT experiment, PXD033591 for the DDA part and PXD033525 for the DIA part.

## Abbreviations

ASCT: Autologous hematopoietic stem cell transplantation
DDA: Data-dependent acquisition mode
DIA: Data independent acquisition mode
HSCT: Hematopoietic stem cell transplantation
LC/MS/MS: Liquid Chromatography – Tandem Mass Spectrometry
LFQ: Label-free quantification
MS: Mass Spectrometry
NF-κB: (Transcription factor) nuclear factor kappa-B
NON-OM: Non-ulcerative oral mucositis
OM: Oral mucositis
TMT: Tandem Mass Tag
TNF-α: Tumor Necrosis Factor alpha
ULC-OM: Ulcerative oral mucositis
