# Supplementary material for: The salivary proteome in relation to oral mucositis in autologous hematopoietic stem cell transplantation recipients: a labelled and label-free proteomics approach
Source: BMC Oral Health. 2023 Jul 7;23:460. doi: 10.1186/s12903-023-03190-w (PMC10329372; doi:10.1186/s12903-023-03190-w)
Supplement: Supplementary file 1 — Additional file 1. Experimental details of the TMT-labelled and Label-Free Quantificationexperiment. [file 12903_2023_3190_MOESM1_ESM.pdf]

Additional file 1: Experimental details

TMT experiment

Preparation and LC/MS/MS analysis. The pooled samples were dried (Speed Vac, Thermo Fisher Scientific) and resuspended in 100 mM TEAB (triethylammonium bicarbonate) buffer and 0.1% SDS. Cysteine residues were reduced with 8 mM TCEP (tris(2-carboxyethyl)phosphine) for 1h at 55 °C subsequently alkylated with 67.5 mM IAA (iodoacetamide) for 30 min at RT. Proteins were digested overnight at 37 °C with 10 µl of 0.2 µg/µl trypsin in 100 mM TEAB + 0.1% TFA.

Each sample was labelled with a unique TMT label in a 1:1 ratio. Forty-one 41 µl of the TMT reagent was added to the sample, vortexed and briefly centrifuged at 14000 rpm before 1h incubation at RT. The reaction was stopped by 15 min incubation at RT with 8 µl of 5% hydroxylamine. After labelling, all samples were mixed, incubated for 15 min at RT and freeze dried (Speed vac, -80 °C).

Before isoelectric focusing samples were resuspended in resuspension buffer (IPG stock solution), vortexed and centrifuged for 1 min at 14000 rpm. Equal volumes of each samples was added for the isoelectric focusing in low resolution 12-well strip with a 3-10 non-linear gradient. Fractions 1 and 7, fractions 2 and 8, etc. were combined to yield 6 samples for LC/MS/MS analysis. Using C18 reversed-phase Zip-Tips (Millipore) glycerol was removed from the samples.

Peptides were chromatographically separated using a 75 µm C18 column (EASY NanoLC system, ThermoFisherScientific, UK) using a three step linear gradient of CAN in 0.1% formic acid. Peptides were eluted at a flow of 300 nl/min for 120 min which ionized by electrospray ionization using an Orbitrap Velos Pro operating under Xcalibur v2.2 (ThermoFisherScientific, UK). Precursor ions were selected for peptide identification and reporter ion fragmentation based on their intensity in an automated data-dependent switching mode and Higher-energy C-trap dissociation (Top 10 method). The MS/MS analyses were conducted using higher than normal collision energy profiles that were chosen based on the m/z ratio and the charge state of the peptide.

Protein identification and quantification. The obtained mgf files were searched against a Homo Sapiens Swiss-Prot generated FASTA file which was downloaded at 21 September 2018. Pig trypsin (P00761) was added and reverse sequences were used as decoys. The MS-GF+ (v2018.04.09) and OMSSA algorithms were used with the following search details in SearchGUI 3.3.4 and PeptideShaker 1.16.29:

- fixed modification: carbamethylation of C
- variable modifications: oxidation of M, TMT 10-plex K, TMT 10-plex of peptide n-term
- enzyme: trypsin, semi-specific, max missed cleavages: 2, fragment ions types b and y

- precursor m/z tolerance: 20 ppm
- fragment m/z tolerance: 0.05 Da
- precursor change 2-4, isotopes 0-1

The validated proteins at a 1% false-negative rate were further analyzed in Reporter 0.7.20 to obtain quantitative ratios using the following settings:

- TMT 10-plex, no reference channel
- Isotope correction: default settings
- Reporter ions: default settings
- Ratio estimation: default settings (minimum of 3 unique peptides, no exclusion of miscleaved peptides)
- Normalization: default settings (median normalization at the peptide and protein level, no stable protein provided)

In the output, the values under ratios were Log_2 transformed and used for data analysis. The principal component analysis (PCA) was performed using the 'princomp' function in RStudio. The heatmap was generated using the heatmap.2 function where the distance was calculated using a correlation measurement (equation 3 as described in Key (2012)) and the colors were scaled per row.

LFQ experiment

Sample preparation. 100 µg of pooled samples from OM and non-OM patients were added to solid urea obtaining a final concentration of 8 M urea. 20 mM HEPES pH 8.0 was added until a volume of 100 µl was reached. First, proteins were reduced through incubation for 30 minutes at 55˚C with 15 mM DTT. Next alkylation was done by incubating for 15 minutes at room temperature in the dark with 30 mM iodoacetamide. Following dilution with 20 mM HEPES pH 8.0 up to 4M urea, proteins were digested with 1 µg lysyl endopeptidase (Wako) (1/100, w/w) for 4 hours at 37°C. Samples were further diluted with 20 mM HEPES pH 8.0 to a final urea concentration of 2 M and proteins were digested with 1 µg trypsin (Promega) (1/100, w/w) overnight at 37˚C. The resulting peptide mixture was purified using OMIX C18 pipette tips (Agilent). The eluent was divided in two aliquots , dried completely by vacuum drying and stored at -20°C until further use.

Off-line high pH reversed phase C18 peptide fractionation. Prior to LC-MS/MS analysis, one aliquot of each sample was fractionated using reversed-phase chromatography at pH 5.5 using an Agilent 1100 series HPLC. A volume corresponding to 50 µg peptide material was trapped for 16 minutes using a reversed-phase trapping column (35 mm x 300 µm I.D., 5 µm beads C18 material (Dr. Maisch, Germany), fritted and packed in-house). Next each sample was separated on an analytical column (150

mm x 250 µm I.D., 3 µm beads C18 material (Dr. Maisch, Germany), fritted and packed in-house) using a 100 min gradient from 100% solvent A (10 mM ammonium acetate, pH 5.5) to 100% solvent B (10 mM ammonium acetate, 70% ACN, pH 5.5) at a constant flow rate of 3 µL/min. Fractions were collected in MS vials per minute over a time interval of 65 min and automatically pooled be restarting the fraction collection cycle every 10 min resulting in 10 pooled fractions. The eluting peptide material was detected at 214 nm and 280 nm. The individual fractions, were dried completely by vacuum drying and stored at -20°C until further use.

LC-MS/MS analysis. Peptides were re-suspended in 20µl of loading solvent (0.1% TFA in water/acetonitrile, 98/2 (v/v)) and 15 µl was injected for LC–MS/MS analysis on an Ultimate 3000 RSLC nano LC (Thermo) in-line connected to a Q Exactive HF mass spectrometer (Thermo). Trapping of the peptides was performed at 10 µl/min for 4 min in loading solvent A on a trapping column (made in-house, 20 mm x 100 µm internal diameter (I.D.), 5 µm beads, C18 Reprosil-HD, Dr. Maisch, Germany). The peptides were separated on a 200 cm µPAC™ column with C18-endcapped functionality (Pharmafluidics, Belgium) kept at a constant temperature of 50°C. Peptides were eluted by a non-linear gradient reaching 33% MS solvent B (0.1% FA in water/acetonitrile (2:8, v/v)) in 105 min, 55% MS solvent B in 145 min and 99% MS solvent B in 150 min followed by a 10-minutes wash at 99% MS solvent B and re-equilibration with MS solvent A (0.1% FA in water) at a constant flow rate of 750 nl/min for 15 min and 300 nl/min for the remaining gradient. The mass spectrometer was operated in data-dependent mode, automatically switching between MS and MS/MS acquisition for the 8 most abundant ion peaks per MS spectrum. Full-scan MS spectra (375-1500 m/z) were acquired at a resolution of 60,000 in the Orbitrap analyzer after accumulation to a target value of 3,000,000. The 8 most intense ions above a threshold value of 8,300 were isolated for fragmentation at a normalized collision energy of 28% after filling the trap at a target value of 100,000 for maximum 120 ms. MS/MS spectra (200-2000 m/z) were acquired at a resolution of 15,000 in the Orbitrap analyzer. A dynamic exclusion of the precursors of 12s was applied.

Data analysis. Data analysis was performed by MaxQuant (version 1.5.6.5) (PMID 19029910) using the Andromeda search engine with default search settings including a false discovery rate set at 1% on both the peptide and protein level. Spectra were searched against the human proteins in the Swiss-Prot database (database release version of January 2019 containing 20,413 human protein sequences, downloaded from www.uniprot.org) and a bacterial database composed out of all protein sequences present in uniprot of all oral species identified by 16S rRNA sequencing in the Human Oral Microbiome Database (HOMD, www.homd.org). The split by taxonomy feature was applied on the phylum level. The mass tolerance for precursor and fragment ions was set to 4.5 and 20 ppm, respectively, during

the main search. Enzyme specificity was set as C-terminal to arginine and lysine (trypsin), also allowing cleavage at arginine/lysine-proline bonds with a maximum of two missed cleavages. Carbamidomethylation of cysteine residues was set as a fixed modification and variable modifications were set to oxidation of methionine residues (to sulfoxides) and acetylation of protein N-termini. Proteins were quantified by the MaxLFQ algorithm integrated in the MaxQuant software (PMID 24942700). Only proteins with at least one unique or razor peptide were retained for identification, while a minimum ratio count of two unique peptides was required for quantification.

The mass spectrometry proteomics data of both the TMT and label-free experiments have been deposited to the ProteomeXchange Consortium [16] via the PRIDE [17] partner repository with the dataset identifiers PXD033603 for the TMT, PXD033591 for the DDA part and PXD033525 for the DIA part.
