# Supplementary material for: The salivary proteome in relation to oral mucositis in autologous hematopoietic stem cell transplantation recipients: a labelled and label-free proteomics approach
Source: BMC Oral Health. 2023 Jul 7;23:460. doi: 10.1186/s12903-023-03190-w (PMC10329372; doi:10.1186/s12903-023-03190-w)
Supplement: Supplementary file 2 — Additional file 2: Supplementary Table 1A and 1B. Listing the up- and down-regulated proteins in the ULC-OM pools versus the NON-OM pools and the involved pathways of these up- and down-regulated proteinsof the TMT-labelled experiment. [file 12903_2023_3190_MOESM2_ESM.pdf]

Additional file 2

Supplementary Table 1A. Up-regulated and down-regulated proteins in the ulcerative oral mucositis (ULC-OM) pools versus the non-oral mucositis (NON-OM) pools of the TMT experiment per timepoint. Z-scores were calculated using log_2 transformed fold changes per time point (up-regulated proteins: Z-score ≥ 2.0, down-regulated proteins: Z-score ≤ 2.0).

**Timepoint (# of proteins in total)**	**Up-regulated**				**Down-regulated**			
	Accession number	Protein name	# of peptides	Log2 fold change	Accession number	Protein name	# of peptides	Log2 fold change
**Baseline (66)**	P23280	Carbonic anhydrase	6	4.35	Q14508	WAP four-disulfide core domain protein 2	2	-3.41
	P60709	Actin, cytoplasmic 1	5	3.61				
**Week 1: 0 – 4 days after HSCT (97)**	P69905	Hemoglobulin subunit alpha	1	1.77	P31151	Protein S100-A7	1	-1.34
	P08779	Keratin, type I cytoskeletal 16	1	2.04	P01859	Immunoglobulin heavy constant gamma 2	2	-1.81
**Week 2: 6 – 11 days after HSCT (97)**	Q9Y6R7	IgGFc-binding protein	5	2.06	P31151	Protein S100-A7	1	-2.76
	O60814	Histone H2B type 1-K	2	1.75	P01859	Immunoglobulin heavy constant gamma 2	2	-2.40
	P02788	Lactotransferrin	21	1.58	Q9UBC9	Small proline-rich protein 3	1	-1.63
	Q96DA0	Zymogen granule protein 16 homolog B	2	1.60				
**Week 3: 13 – 18 days after HSCT (97)**	Q9Y6R7	IgGFc-binding protein	5	2.13	P07108	Acyl-CoA-binding protein	1	-1.83
	P03973	Antileukoproteinase	7	2.60	Q6UWP8	Suprabasin	1	-1.73
	P61626	Lysozyme C	4	3.06				
	P15515	Histatin-1	2	2.27				
**3 months after HSCT (97)**	P31025	Lipocalin-1	7	2.30	P01859	Immunoglobulin heavy constant gamma 2	2	-2.25
	P02812	Basic salivary proline-rich protein 2	11	2.79	P59665	Neutrophil defensin-1	3	-2.45
	P01782	Immunoglobulin heavy variable 3-9	1	2.55				
	P06870	Kalikrein-1	5	1.72				

Supplementary Table 1B. Involved pathways of the up-regulated and down-regulated proteins shown in Supplementary Table 1A.

Timepoints	Up-regulated	Down-regulated
**Baseline**	Metabolism: reversible hydration of carbon dioxide<br>Vesicle mediated transport/ cell-cell communication/ signal transduction/ gene expression	Protease inhibitor
**Week 1: 0 – 4 days after HSCT**	Transport of small molecules: O_2/CO_2 exchange<br>Developmental biology: formation of the cornified envelop	Innate immune system:<br>- Antimicrobial peptides<br>- Activation and regulation of complement cascade<br>- FC gamma receptor dependent phagocytosis
**Week 2: 6 – 11 days after HSCT**	Maintenance of mucosal structures<br>Gene expression/ transcription/ DNA repair/ cell cycle<br>Immune system/ amyloid fiber formation<br>Carbohydrate binding/ retina homeostasis	Innate immune system:<br>- Antimicrobial peptides<br>- Activation and regulation of complement cascade<br>- FC gamma receptor dependent phagocytosis<br>Developmental biology: formation of the cornified envelop
**Week 3: 13 – 18 days after HSCT**	Maintenance of mucosal structures<br>Innate immune system:<br>- Antimicrobial peptides<br>- Activation and regulation of complement cascade<br>- FC gamma receptor dependent phagocytosis	Mitochondrial fatty acid beta-oxidation<br>Suprabasin: unknown,<br>Up-regulated in differentiating keratinocytes
**3 months after HSCT**	Transport of fatty acids<br>Basic salivary proline-rich protein 2: unknown<br>Adaptive immune system:<br>- B-cell receptor signaling<br>- Classical antibody-mediated complement activation<br>Metabolism of proteins: regulation of insulin-growth factor transport and uptake	Innate immune system:<br>- Antimicrobial peptides<br>- Activation and regulation of complement cascade<br>- FC gamma receptor dependent phagocytosis
