# Supplementary material for: The salivary proteome in relation to oral mucositis in autologous hematopoietic stem cell transplantation recipients: a labelled and label-free proteomics approach
Source: BMC Oral Health. 2023 Jul 7;23:460. doi: 10.1186/s12903-023-03190-w (PMC10329372; doi:10.1186/s12903-023-03190-w)
Supplement: Supplementary file 3 — Additional file 3: Supplementary Tables, 2, 3, 4 and 5 of the DIA experiment. Listing the significant enriched biological processes GO-terms of the significantly regulated proteins in the NON-OM samples across all timepoints; the significantly expressed proteins of the two-way ANOVA of the DIA experiment; the differently expressed proteins during the hospitalization period or outside the hospitalization period); and the top 5 of the significantly enriched biological processes GO-terms of those proteins listed in Supplementary Table 4. [file 12903_2023_3190_MOESM3_ESM.pdf]

Additional file 3

Supplementary Table 2. Significantly enriched GO terms for the biological processes of the significantly regulated proteins (n=12, Table 1) in the non-oral mucositis (NON-OM) samples across all timepoints in the Data Independent Acquisition (DIA) experiment.

Term ID	Term name	Adjusted p-value nOM*
GO:0002250	Adaptive immune response	0.0008
GO:0002443	Leukocyte mediated immunity	0.0035
GO:0006955	Immune response	0.0056
GO:0002252	Immune effector process	0.0317
GO:0002449	Lymphocyte mediated immunity	0.0318
GO:0002460	Adaptive immune response based on somatic recombination of immune receptors built from immunoglobulin superfamily domains	0.0336
GO:0045321	Leukocyte activation	0.0370
GO:0150078	Positive regulation of neuroinflammatory response	0.0378

*Bonferroni correction

Supplementary Table 3. Significantly different expressed proteins of the two-way ANOVA between the different conditions (ulcerative oral mucositis (ULC-OM) vs non-oral mucositis (NON-OM)), timepoints and the interaction (OM*timepoint) in the Data Independent Acquisition (DIA) experiment. The significant values ($p<0.05$) are shown in green cells.

Accession number	Protein name	-Log p-value OM	-Log p-value timepoint	-Log p-value interaction
A0A3E4XAZ8*	Enolase*	0.627	1.501	1.349
O43278	Kunitsz-type protease inhibitor 1	0.933	1.897	1.609
O60235	Transmembrane protease serine 11D	1.925	1.781	1.540
O95834	Echinoderm microtubule-associated protein-like 2	0.295	0.322	1.302
P00390	Glutathione reductase, mitochondrial	0.042	0.425	1.387
P01040	Cystatin-A	1.354	2.373	2.278
P02812	Basic salivary proline-rich protein 2	0.084	0.635	2.421
P04259	Keratin, type II cytoskeletal 6B	2.011	0.695	1.321
P04406	Glyceraldehyde-3-phosphate dehydrogenase	0.144	2.266	1.681
P05120	Plasminogen activator inhibitor 2	0.794	3.329	1.517
P05387	60S acidic ribosomal protein P2	0.764	1.633	2.017
P06454	Prothymosin alpha	1.106	0.321	1.578
P08727	Keratin, type I cytoskeletal 19	0.890	1.458	1.506
P13489	Ribonuclease inhibitor	0.004	0.058	1.731
P14618	Pyruvate kinase PKM	0.946	1.035	2.323
P15309	Prostatic acid phosphatase	1.003	1.865	1.731
P15531	Nucleoside diphosphate kinase A	0.766	1.027	1.362
P18510	Interleukin-1 receptor antagonist protein	2.788	7.747	1.517

P20618	Proteasome subunit beta type-1	0.258	0.077	2.174
P22626	Heterogeneous nuclear ribonucleoproteins A2/B1	0.149	3.386	1.632
P24534	Elongation factor 1-beta	0.163	1.227	1.704
P30520	Adenylosuccinate synthethase isozyme 2	1.752	4.813	1.358
P30838	Aldehye dehydrogenase, dimeric NADP-preferring	2.633	2.649	1.586
P40394	All-trans-retinol dehydrogenase [NAD(+)] ADH7	0.257	0.353	1.383
P49720	Proteasome subunit beta type-3	1.072	0.913	1.890
P52209	6-phosphogluconate dyhydrogenase, decarboxylating	0.810	0.462	1.646
P52907	F-actin-capping proteins subunit alpha-1	0.384	1.531	1.468
P84090	Enhancer of rudimentary homolog	1.695	0.268	1.531
Q16651	Prostasin	0.431	0.685	1.398
Q6XQN6	Nicotinate phosphoribosyltransferase	0.425	0.206	1.387
Q86X29	Lipolysis-stimulated lipoprotein receptor	2.019	3.098	1.550
Q96G03	Phosphoglucomutase-2	2.797	2.848	1.768
Q9UHA7	Interleukin-36 alpha	0.174	0.540	1.523
S2ZMZ1*	Galactose-1-phophate uridylyltransferase*	0.277	1.027	1.374

*microbial proteins

Supplementary Table 4. Differently expressed proteins during hospitalization or outside the hospitalization period (baseline, 3 months and 12 months), the red dots/proteins in the volcano plot (Figure 12; Data Independent Acquisition (DIA) experiment).

**Protein accession number**	**Protein name**	**-log 10 adjusted p-value***	**Log 2 fold change**
**Up-regulated during hospitalization (week 1, 2 and 3)**			
Q6UW32	Insulin growth factor-like family member 1	-1.958	9.522
P22528	Cornifin-B	-1.898	13.936
P35321	Cornifin-A	-1.640	10.063
P22532	Small proline-rich protein 2D	-1.630	8.369
P35326	Small proline-rich protein 2A	-1.559	12.694
P09237	Matrilysin	-1.439	4.505
P01011	Alpha-1-antichomotrypsin	-1.318	6.686
O43505	Beta-1,4-glucuronyltransferase 1	-1.314	3.308
Q12805	EGF-containing fibulin-like extracellular matrix protein 1	-1.289	2.204
Q9UBC9	Small proline-rich protein 3	-1.278	6.663
P19835	Bile salt-activated lipase	-1.249	1.527
P19957	Elafin	-1.207	5.158
P29508	Serpin B3	-1.146	16.672
P49862	Kallikrein-7	-1.102	2.183
Q15782	Chitinase-3-like protein 2	-1.065	7.549

P14555	Phospholipase A2, membrane associated	-1.045	1.467
P0DJI8	Serum amyloid A-1 protein	-1.017	2.529
Q96QR1	Secretoglobin family 3A member 1	-1.009	2.616
**Down-regulated during hospitalization (up-regulated at baseline, 3 months and 12 months)**			
P25815	Protein S100-P	1.524	6.695
P24158	Myeloblastin	1.422	4.384
A0A0A0MS15	Immunoglobulin heavy variable 3-49	1.393	1.411
P01591	Immunoglobulin J chain	1.385	9.241
Q9H4A4	Aminopeptidase B	1.377	2.072
P01619	Immunoglobulin kappa variable 3-20	1.304	7.875
P14780	Matrix metalloproteinase-9	1.291	5.769
P69905	Hemoglobin subunit alpha	1.248	1.738
P49913	Cathelicidin antimicrobial peptide	1.204	2.908
P31946	14-3-3 protein beta/alpha	1.197	2.443
P50552	Vasodilator-stimulated phosphoprotein	1.181	2.861
P06753	Tropomyosin alpha-3 chain	1.171	4.354
C8PSJ9	Unknown	1.159	6.837
P01876	Immunoglobulin heavy constant alpha 1	1.139	7.358
P0DOX2	Immunoglobulin alpha-2 heavy chain	1.105	5.335
A0A0B4J1X5	Immunoglobulin heavy variable 3-74	1.071	5.065
P01834	Immunoglobulin kappa constant	1.061	7.495
P06312	Immunoglobulin kappa variable 4-1	1.029	5.965
P08246	Neutrophil elastase	1.026	3.389

*Bonferroni correction

Supplementary Table 5. Top 5 of the significantly enriched GO terms for the biological processes of the significantly regulated proteins (n=37, Supplementary Table 4, Figure 12) during or outside the hospitalization period of the Data Independent Acquisition (DIA) experiment.

Term ID	Term name	Adjusted p-value nOM*
**Up-regulated during hospitalization (week 1, 2 and 3)**		
GO:0031424	Keratinization	1.050×10^{-6}
GO:0018149	Peptide cross-linking	1.050×10^{-5}
GO:0030216	Keratinocyte differentiation	6.184×10^{-5}
GO:0008544	Epidermis development	1.273×10^{-4}
GO:0009913	Epidermal cell differentiation	3.788×10^{-4}
**Down-regulated during hospitalization (up-regulated at baseline, 3 months and 12 months)**		
GO:0006959	Humoral immune response	1.975×10^{-8}
GO:0019730	Antimicrobial humoral response	2.084×10^{-7}
GO:0019731	Antibacterial humoral response	5.434×10^{-7}
GO:0042742	Defense response to bacterium	2.933×10^{-6}
GO:0009617	Response to bacterium	4.285×10^{-4}

*Bonferroni correction
